# ^89^Zr anti-CD44 immuno-PET monitors  CD44 expression on splenic myeloid cells and HT29 colon cancer cells

**DOI:** 10.1038/s41598-021-83496-3

**Published:** 2021-02-16

**Authors:** Jin Won Park, Kyung-Ho Jung, Jin Hee Lee, Seung Hwan Moon, Young Seok Cho, Kyung-Han Lee

**Affiliations:** 1 Scripps Korea Antibody Institute, 1, Kangwondeahak-gil, Chuncheon-si, Gangwon-do Korea; 2 Department of Nuclear Medicine, Samsung Medical Center, 50 Ilwon-dong, Gangnam-gu, Seoul, Korea; 3 Department of Health Sciences and Technology, SAIHST, Sungkyunkwan University School of Medicine, Seoul, Korea

**Keywords:** Biotechnology, Cancer, Immunology, Molecular biology, Stem cells

## Abstract

CD44 is a cell-surface glycoprotein involved in cell–cell interaction, adhesion, and migration. CD44 is found on colon cancer cells and on immune cells. Previous studies of ^89^Zr PET imaging of CD44 have relied on an anti-human antibody (Ab), which can influence biodistribution in murine models. In this study, we used an Ab that cross-reacts with both human and mouse origin CD44 of all isoforms to unveil the type of leukocyte responsible for high splenic anti-CD44 uptake and investigate how its regulation can influence tumor immuno-PET. The Ab was site-specifically labeled with ^89^Zr-deferoxamine on cysteine residues. ^89^Zr-anti-CD44 demonstrated high-specific binding to HT29 human colon cancer cells and monocytic cells that showed CD44 expression. When ^89^Zr-anti-CD44 was administered to Balb/C nude mice, there was remarkably high splenic uptake but low SNU-C5 tumor uptake (1.2 ± 0.7%ID/g). Among cells isolated from Balb/C mouse spleen, there was greater CD44 expression on CD11b positive myeloid cells than lymphocytes. In cultured monocytic and macrophage cells, LPS stimulation upregulated CD44 expression and increased ^89^Zr-anti-CD44 binding. Similarly, normal Balb/C mice that underwent lipopolysaccharide (LPS) stimulation showed a significant upregulation of CD44 expression on splenic myeloid cells. Furthermore, LPS treatment stimulated a 2.44-fold increase of ^89^Zr-anti-CD44 accumulation in the spleen, which was attributable to splenic myeloid cells. Finally, in Balb/C nude mice bearing HT29 tumors, we injected ^89^Zr-anti-CD44 with greater Ab doses to reduce binding to splenic cells. The results showed lower spleen uptake and improved tumor uptake (2.9 ± 1.3%ID/g) with a total of 300 μg of Ab dose, and further reduction of spleen uptake and greater tumor uptake (5.7 ± 0.0%ID/g) with 700 μg Ab dose. Thus, using an ^89^Zr labeled Ab that cross-reacts with both human and mouse CD44, we demonstrate that CD44 immuno-PET has the capacity to monitor CD44 regulation on splenic myeloid cells and may also be useful for imaging colon tumors.

## Introduction

CD44 is a multifunctional, non-kinase, single-pass transmembrane glycoprotein involved in cell–cell and cell-extracellular matrix interactions^1^. Malignant cells expressing CD44 are associated with tumor initiation and tumorsphere formation capacity that leads to cancer progression and poor patient outcome. Indeed, CD44 is a major marker of cancer stemness as characterized by self-renewal capacity, epithelial-mesenchymal transition, and treatment resistance^1^. CD44 thus provides an attractive target for cancer treatment^2–4^, and radiolabeled anti-CD44 antibodies (Abs) are being investigated for targeted imaging^5–9^, as well as radio-immunotherapy of solid tumors^10,11^.

However, CD44 is also expressed on leukocytes, where it plays roles in cell mobilization and function^12^. In the body, the spleen serves as the largest secondary lymphoid organ and hosts large numbers of phagocytic myeloid cells as well as lymphocytes^13^. Immune staining confirmed CD44 expression on leukocytes in the human spleen and facilitated its micro-anatomical compartmentalization^14^. Among many different splice variants of CD44, certain variants such as CD44v6 is reported to have a more restricted expression in a subset of epithelial tissues and mostly epithelial tumors. In contrast, Abs directed against the constant domain of CD44 is likely to target other tissues such as splenic leukocytes in addition to tumor cells. Immuno-PET using such Abs could thus yield useful information regarding splenic immune responses^17,18^.

Among splenic leukocytes, myeloid cells that include monocytes/macrophages and neutrophils have discrete immune functions with central roles in cellular stress, foreign material removal, and immune response regulation^15^. Myeloid cells become activated and upregulated in function by inflammatory stimuli, and lipopolysaccharide (LPS) stands out as a major stimulator through the classical activation pathway^16^. LPS released from bacteria enters the circulation to stimulate the immunologic system by activating myeloid cells. Indeed, activation response to LPS is one of the best characterized pathogen-associated molecular patterns that cause phagocytic cells to switch to an inflammatory phenotype without leaving the tissue.

A previous study using an ^89^Zr-labelled anti-human CD44 Ab observed high uptake in human cancer xenografts with low spleen uptake in mice but found remarkably high splenic uptake in non-human primates^7^. Therefore, in vivo biodistribution and imaging findings in human cancer-bearing mice that better predicts the results in human subjects will be benefited from the use of an Ab that cross-reacts with both murine and human CD44.

This aim of this study was to use an Ab that reacts with all CD44 isoforms of both human and mouse origin to unveil how splenic leukocyte activation affects spleen uptake of ^89^Zr-anti-CD44 and to dissect the leukocyte type responsible for this effect. We further investigated how regulating the magnitude of splenic leukocyte uptake by total Ab dose can influence tumor accumulation.

## Results

### Deferoxamine (DFO) conjugation and site-specific ^89^Zr labeling of anti-CD44 Ab

**Figure 1:**
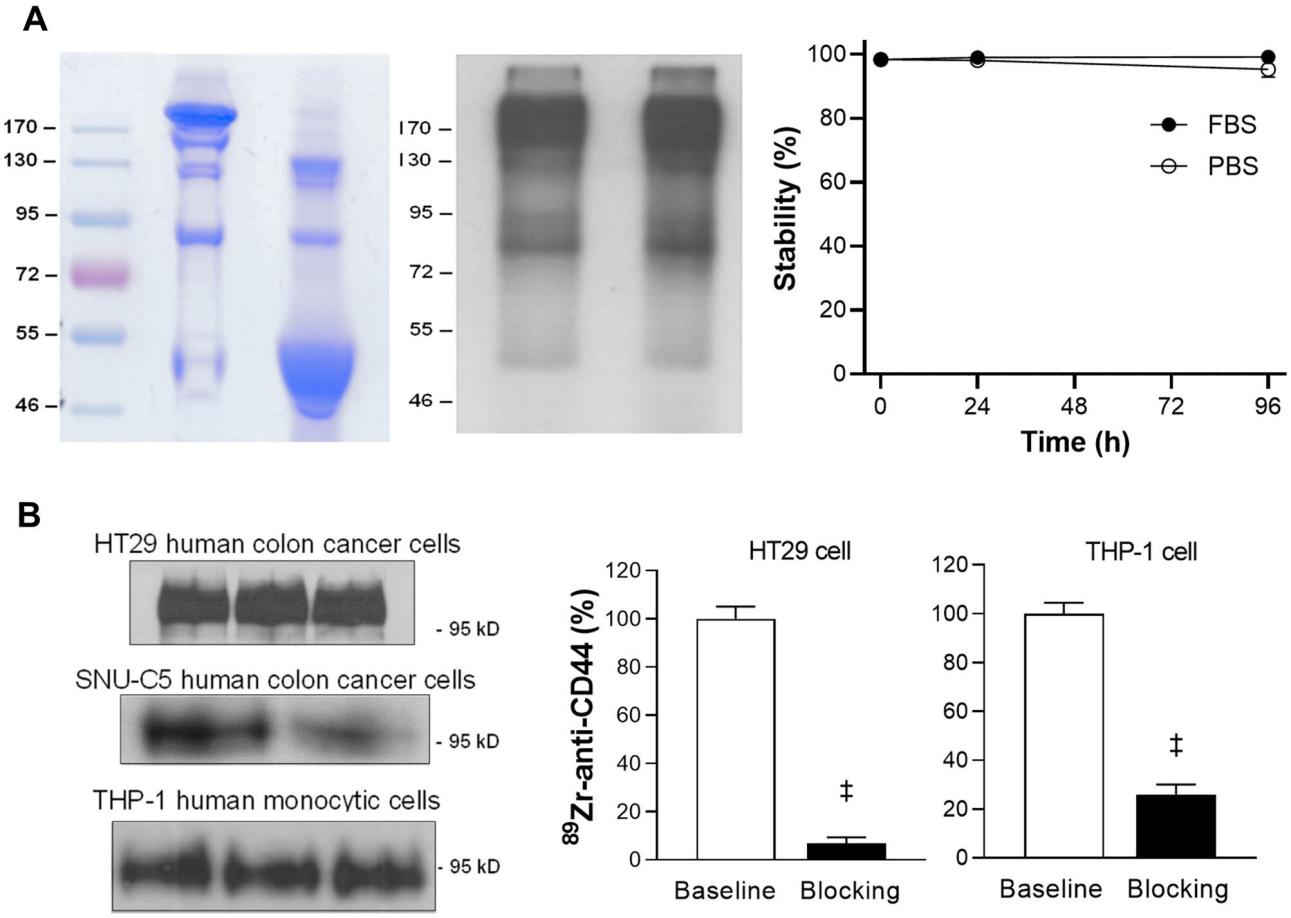
^89^Zr-anti-CD44 preparation, stability, and CD44-specific binding. (**A**) Non-reduced SDS polyacrylamide gel electrophoresis (PAGE) of unmodified and deferoxamine-conjugated anti-CD44 (left). Autoradiograph of ^89^Zr-anti-CD44 on native PAGE (middle), and in vitro stability in PBS and FBS (n = 4) assessed by radio-instant thin layer chromatography (right). (**B**) CD44 expression on HT29 human colon cancer cells and THP-1 human monocytic cells (left). Blots were cropped to combine into a single figure (see supplementary Fig. S1 for the full-length blots). CD44-specific cell binding of ^89^Zr-anti-CD44 that was blocked by excess (500 nM) cold anti-CD44 (right). Bars are mean ± S.D. of % bound activity from triplicate samples per group. ‡*P* > 0.001.

IM7 Ab was site-specifically conjugated with ^89^Zr on cysteine residues in a straightforward manner. The Ab was first reduced through reaction with tris(2-carboxyethyl)phosphine (TCEP) and then underwent conjugation with deferoxamine (DFO)-maleimide. Non-reduced SDS-PAGE analysis demonstrated complete reduction of target disulfide bonds of the Ab by TCEP, which remained reduced after DFO-conjugation (Fig. 1A). ^89^Zr radiolabeling of deferoxamine-conjugated anti-CD44 was reproducible, with a labeling efficiency of 80%. Radiochemical purity was > 99%, and the specific activity was 59.2 MBq/mg. Autoradiography of column-eluted ^89^Zr-anti-CD44 showed a clear radioactive band at the expected 170 kD region (Fig. 1A). Radiochemical stability by radio-instant thin layer chromatography (radio-iTLC) analysis showed that the radiolabel was more than 95% intact after up to 96 h incubation in 50% FBS or PBS (Fig. 1A).

### CD44 expression on cancer cells and monocytic cells and specific ^89^Zr-anti-CD44 binding

Western blots confirmed clear protein bands of CD44 expression on human HT29 and SNU-C5 human colon cancer cells and THP-1 human monocytic cells (Fig. 1B). Cell binding experiments demonstrated that ^89^Zr-anti-CD44 binding to HT29 cells was almost completely blocked to 6.8 ± 2.6% of controls by excess cold anti-CD44, confirming CD44-specific binding (Fig. 1B). Binding to THP-1 cells was also substantially blocked by excess cold anti-CD44 to 25.9 ± 4.0% of controls (Fig. 1B).

### ^89^Zr-anti-CD44 biodistribution in normal mice and CD44 expression on splenic leukocytes

**Figure 2:**
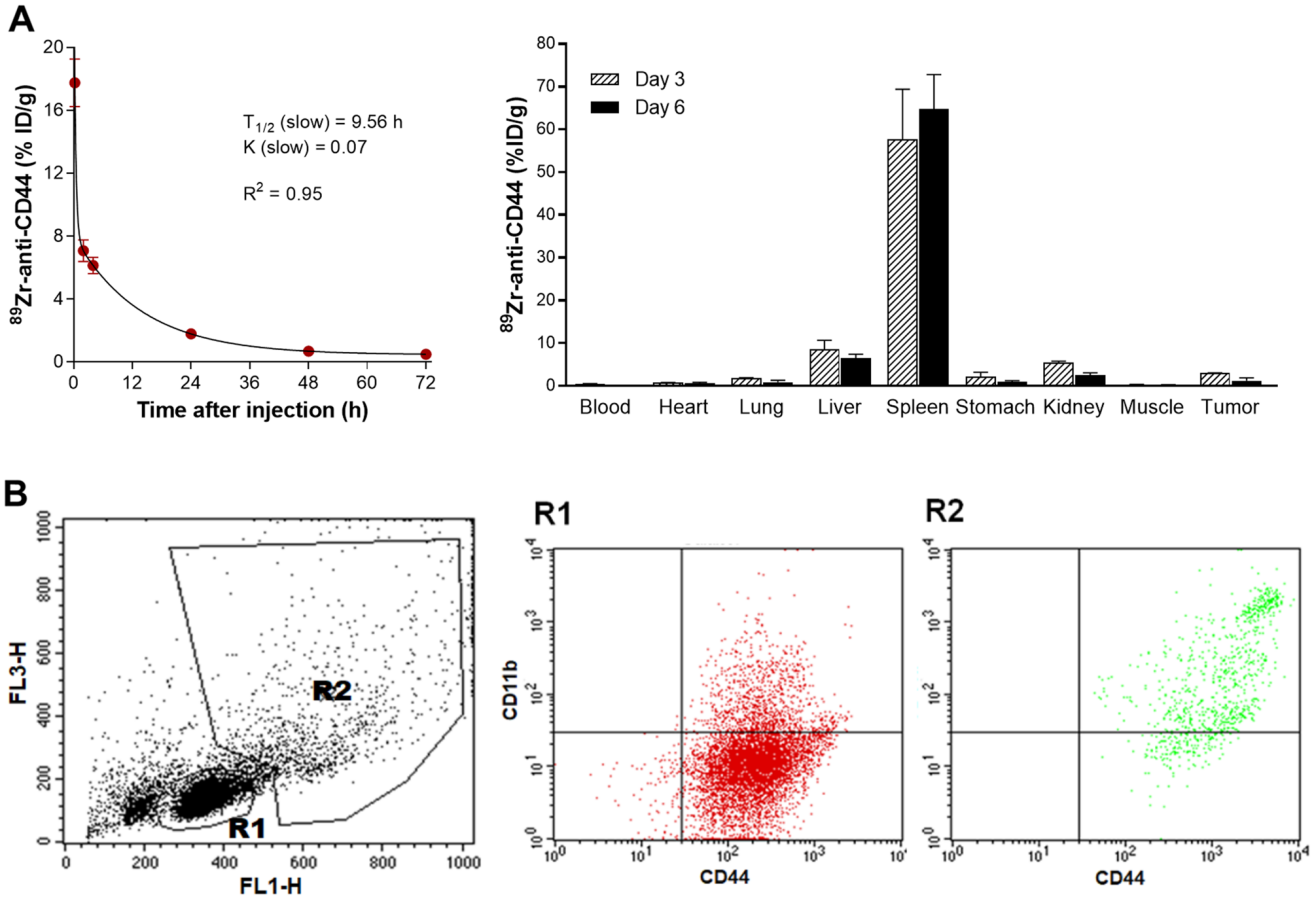
^89^Zr-anti-CD44 pharmacokinetics and biodistribution in mice, and CD44 expression on leukocytes from mouse spleen. (**A**) ^89^Zr-anti-CD44 pharmacokinetics in normal Balb/c mice (n = 5) and biodistribution in SNU-C5 tumor-bearing Balb/c nude mice (n = 4) after intravenous injection. Data are mean ± S.D. of % ID/g. (**B**) CD44 and CD11b expression on leukocytes isolated from the spleen of a normal Balb/c mouse. Single-cell suspensions were obtained by mincing the spleen. Representative flow cytometry forward and side scatter analysis plots (left) demonstrate the R1 region that corresponds to lymphocytes and R2 region that corresponds to myeloid cells. Flow cytometry with FITC-anti-CD44 and APC-anti-CD11b (right) reveal that cells of the R2 region are CD11b + /CD44 + .

When ^89^Zr-anti-CD44 was intravenously injected into normal Balb/c mice, blood pharmacokinetic analysis using a two-phase decay model showed a slow K value of 0.07 and slow circulating half-life of 9.6 h (Fig-2A). Biodistribution data of ^89^Zr-anti-CD44 in SNU-C5 tumor-bearing Balb/c nude mice at 3- and 6-days post-injection are shown in Fig. 2A and Supplementary Table 1. The results revealed remarkably high uptake in the spleen that reached 64.8 ± 8.0 injected dose per gram tissue (%ID/g) at day 6 (Fig. 2A). This was followed by modest uptake in the liver (6.4 ± 1.0%ID/g). There was low uptake in the kidney, myocardium, lung, stomach, and muscle. Blood activity was quite low (0.12 ± 0.0%ID/g). Tumor uptake in these animals was disappointingly low at 1.2 ± 0.7%ID/g (Fig. 2A).

To investigate the cells responsible for the high ^89^Zr-anti-CD44 uptake, leukocytes were isolated from the spleen of normal mice. Flow cytometry with forward and side scatter plots displayed regions R1 and R2 that correspond to lymphocytes and myeloid cells, respectively (Fig. 2B). Dot plots using FITC-anti-CD44 and APC-CD11b revealed that R2 cells were CD11b + /CD44 + , indicating myeloid cells (Fig. 2B).

### Effects of LPS stimulation on cultured macrophages and monocytic cells

**Figure 3:**
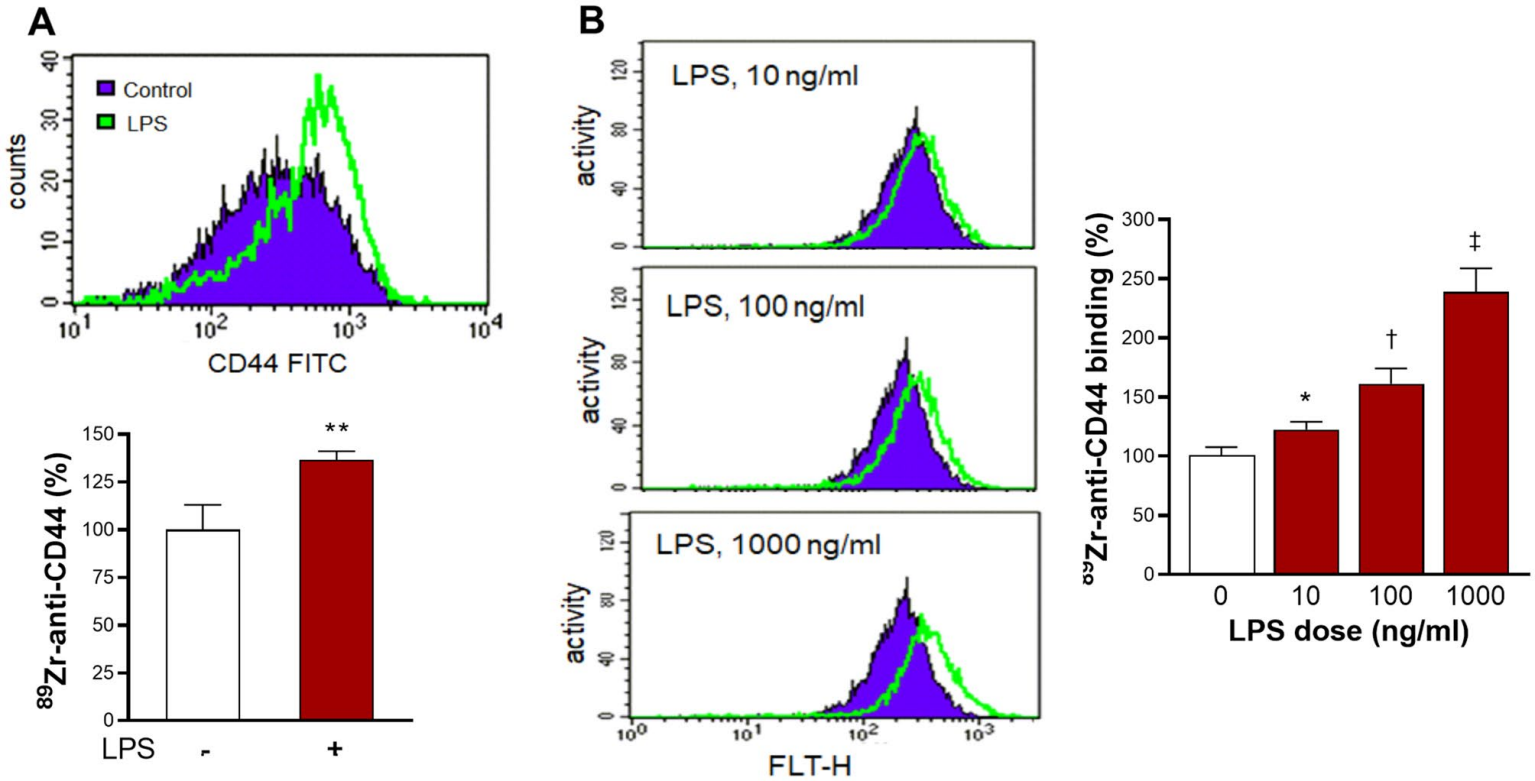
LPS stimulates CD44 expression and ^89^Zr-anti-CD44 uptake in cultured monocytic cells. (**A**,**B**) RAW264.7 murine macrophages (**A**) and THP-1 human monocytic cells (**B**) show increased CD44 expression and ^89^Zr-anti-CD44 uptake after LPS stimulation. Purple regions and green lines indicate control and LPS-stimulated cells, respectively. Bars are mean ± S.D. of % activity obtained from triplicate samples. ^*^P > 0.05; ^**^P > 0.01; ^†^*P* > 0.005; ^‡^*P* > 0.001, compared to untreated controls.

When we tested the effect of LPS on cultured macrophage/monocytic cells, RAW264.7 macrophages showed that LPS significantly stimulated CD44 expression and increased ^89^Zr-anti-CD44 binding to 136.6 ± 4.6% of that of controls (Fig. 3A). Similarly, THP-1 monocytic cells showed that LPS stimulation dose-dependently elevated CD44 expression and ^89^Zr-anti-CD44 binding. The latter reached 238.9 ± 19.9% of untreated controls by 1 μM LPS (Fig. 3B).

### Effect of LPS treatment of mice on CD44 expression of splenic leukocytes

**Figure 4:**
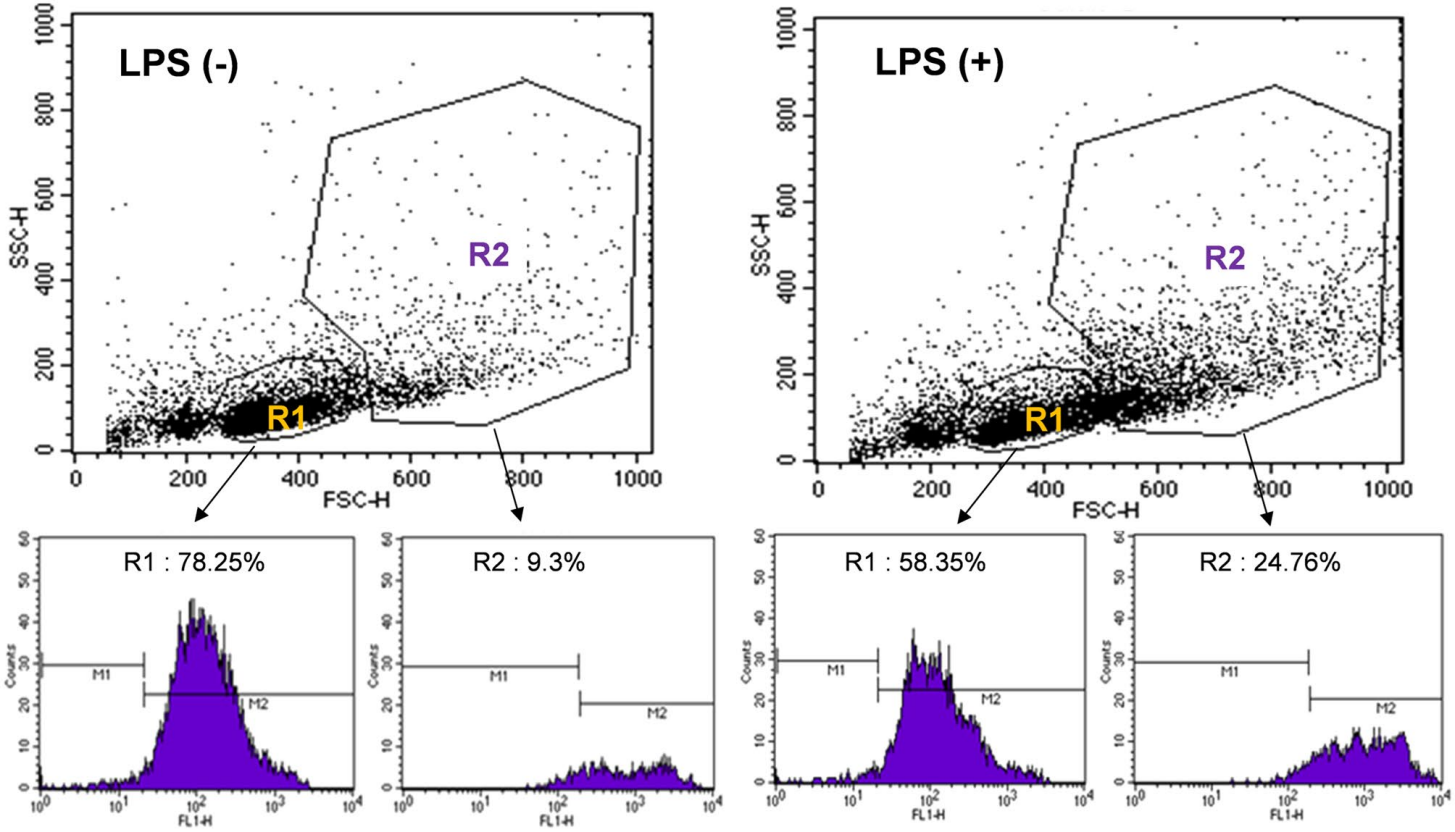
CD44 expression on leukocytes isolated from spleen of mice with or without LPS treatment. Representative forward and side scatter analysis plots show an increase of cells in R2 after mice were treated with LPS (top). Flow cytometry histograms using FITC-anti-CD44 show that LPS stimulates a substantial increase of CD44 expression on cells in R2 but not R1 (bottom).

Flow cytometry histograms of R1 and R2 region splenic leukocytes using FITC-anti-CD44 showed that, although there was a greater number of CD44-positive cells in region R1, the level of CD44 expression was substantially higher for cells in region R2 (Fig. 4). This implied greater contribution of myeloid cells to high splenic ^89^Zr-anti-CD44 uptake.

When we treated normal mice with daily intraperitoneal injection with LPS for 3 days, CD44-positive splenic leukocytes in region R2 increased substantially from 9.3% to 24.8%. In contrast, cells in region R1 showed a decrease of CD44-positivity from 78.3% to 58.3% (Fig. 4). This indicates that LPS simulates CD44 expression in splenic myeloid cells.

### Effects of LPS on ^89^Zr-anti-CD44 PET and biodistribution in normal mice

**Figure 5:**
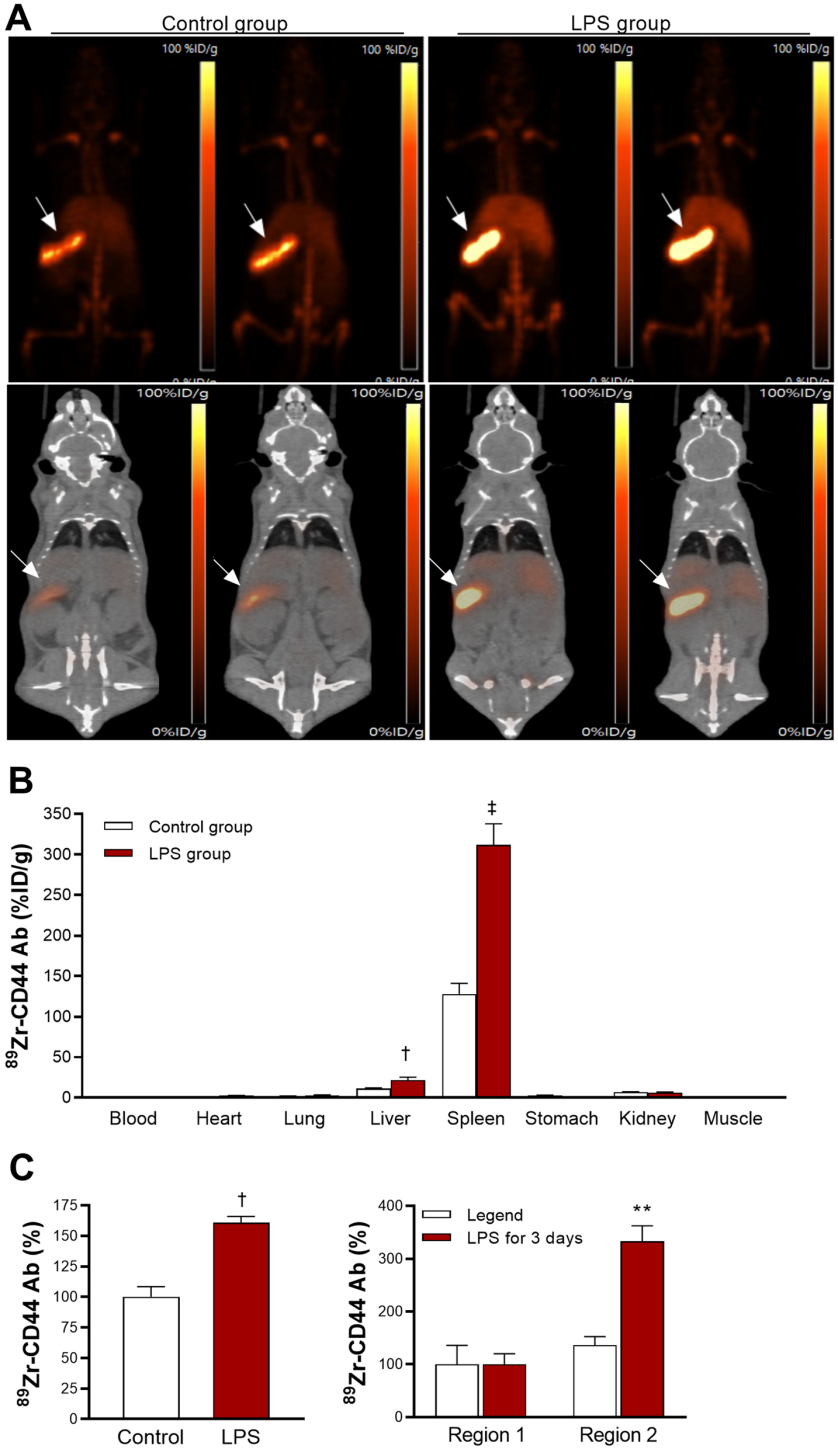
PET/CT imaging, biodistribution, and splenic cell binding of ^89^Zr-anti-CD44. (**A**) Representative maximum intensity projection (MIP) images (top) and coronal (bottom) PET/CT images of two wild type mice per group after daily injection for 3 days with saline (control) or 50 μg/kg LPS. ^89^Zr-anti-CD44 was injected on the second day, and imaging was performed 4 days later. Arrows indicate splenic uptake. (**B**) Biodistribution in saline- and LPS-treated mice immediately after PET/CT. (**C**) Effects of LPS on ^89^Zr-anti-CD44 uptake in leukocytes obtained from spleens of normal naive mice. On the left are unsorted splenic leukocytes, and on the right are cells from R1 and R2. Bars are mean ± S.D. of %ID/g obtained from 4 animals per group (**B**) or % activity obtained from triplicate samples (**C**). ^**^P > 0.01; ^†^*P* > 0.005; ^‡^*P* > 0.001, compared to controls.

On in vivo LPS treatment experiments, PET/CT imaging of wild type Balb/c mice at baseline demonstrated high splenic uptake of ^89^Zr-anti-CD44, and this was substantially increased by following LPS treatment (Fig. 5A).

Biodistribution data confirmed remarkably high splenic uptake at baseline that was even greater than that observed in Balb/c nude mice (127.8 ± 13.5%ID/g; Fig. 5B; Supplementary Table 1). This was thought to reflect differences in the characteristics of splenic leukocytes between the two mouse strains^19^. In addition, since the nude mice had tumors that were substantially larger than the spleen, this took up a significant portion of the administered Ab dose, leaving somewhat less to be taken up by the spleen. In normal mice, splenic uptake was further increased 2.44-fold after LPS treatment (*P* < 0.001; Fig. 5B). Uptake in the liver was slightly increased by LPS treatment, whereas uptake in other organs were not affected.

When ^89^Zr-anti-CD44 uptake in splenic cells isolated from normal mice were compared, unsorted splenic leukocytes showed uptake increased to 160.7 ± 5.0% of that of controls by LPS stimulation (*P* = 0.003; Fig. 5C). R2 region myeloid cells sorted by flow cytometry showed a greater increase of uptake by LPS stimulation to 333.17 ± 29.1% of that of controls (*P* = 0.008), whereas there was no change in uptake for R1 region lymphocytes (Fig. 5C).

### Effects of Ab dose on ^89^Zr-anti-CD44 PET imaging and HT29 tumor uptake

**Figure 6:**
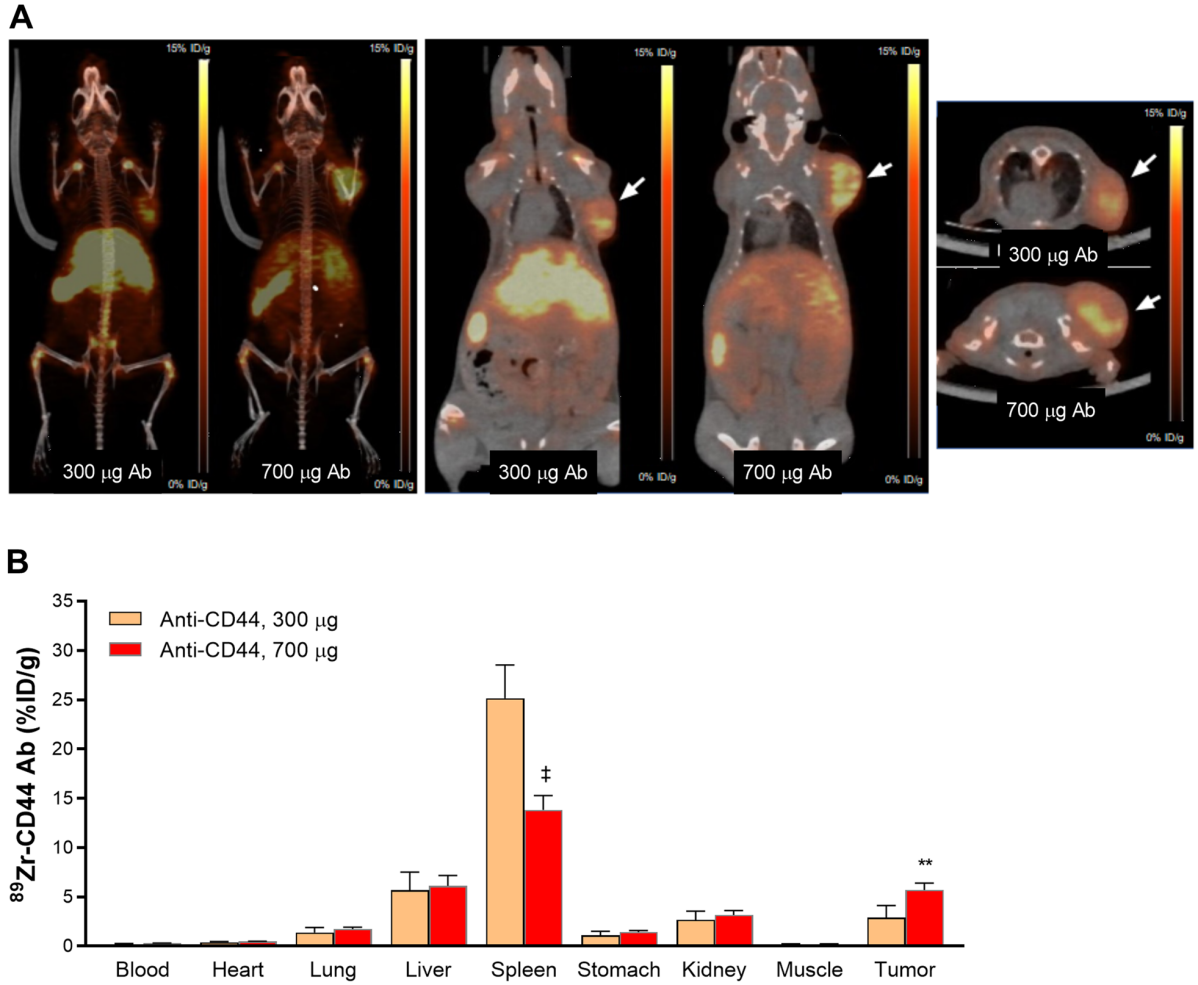
PET/CT imaging and biodistribution of ^89^Zr-anti-CD44 in tumor bearing mice. (**A**) Representative MIP (left), coronal (middle), and transaxial (right) PET/CT images of HT29 tumor- bearing Balb/c nude mice at 4 days after injection of ^89^Zr-anti-CD44 with 300 or 700 ng total Ab doses. Arrows indicate tumor uptake. (**B**) Biodistribution at 4 days after injection of ^89^Zr-anti-CD44 with 300 or 700 ng total Ab doses. Bars are mean ± S.D. of %ID/g obtained from four animals per group. ^**^P > 0.01; ^‡^*P* > 0.001.

Finally, we investigated ^89^Zr-anti-CD44 biodistribution in mice administered with elevated Ab doses. This was based on the notion that elevated Ab doses might reduce splenic cell binding of the radiotracer, thus allowing greater delivery to and accumulation by the tumor. When ^89^Zr-anti-CD44 was injected into HT29 tumor-bearing Balb/c nude mice with a total Ab dose of 300 μg, PET/CT images at day 4 demonstrated clearly visible HT29 tumors and showed splenic uptake that appeared lower than previous observed with low Ab dose (Fig. 6A). When ^89^Zr-anti-CD44 was injected with a total Ab dose of 700 μg, PET images showed further reduced spleen uptake and further increase of HT29 tumor uptake (Fig. 6A).

Biodistribution data confirmed the PET findings (Fig. 6B and Supplementary Table 1). Thus, ^89^Zr-anti-CD44 uptake in the spleen was 25.2 ± 3.4%ID/g with an Ab dose of 300 μg, and further reduced to 13.8 ± 1.4%ID/g with an Ab dose of 700 μg (*P* < 0.001; Fig. 6B). HT29 tumor uptake that was 2.9 ± 1.3%ID/g with an Ab dose of 300 μg was increased two-fold with an Ab dose of 700 μg to 5.7 ± 0.0%ID/g (*P* < 0.01; Fig. 6B). Blood level showed a trend for slight increase by 700 μg Ab dose, while uptake in at other major organs was not influenced.

## Discussion

In this study, we developed an immuno-PET technique that can noninvasively image CD44 status in target tissues of living bodies. The monoclonal rat IgG2b isotype Ab used in this study called IM7 is widely used for CD44 research^20–22^. Labeling of IM7 with ^89^Zr (3.3-days physical half-life) allowed delayed imaging required for Ab probes with long circulating times. The DFO-maleimide conjugation technique that we used leads to cysteine-specific conjugation as a way of tailoring the location of ^89^Zr attachment for PET imaging^23,24^. SDS PAGE analysis indicted that TCEP treatment led to site-specific reduction and deferoxamine conjugation of IM7. This offers advantages for immuno-PET through greater radioprobe homogeneity and immuno-reactivity compared to nonspecific radiolabeling approaches. ^89^Zr-anti-CD44 showed target-specific binding to CD44-positive HT29 colon cancer cells that was almost completely blocked by excess cold Ab, indicating high immuno-reactivity.

IM7 recognizes an epitope common to alloantigens and all isoforms of CD44^25^. Among many different splice variants of CD44, certain variants such as CD44v6 is reported to have a more restricted expression in a subset of epithelial tissues and mostly epithelial tumors. In contrast, Abs directed against the constant domain of CD44 is likely to target other tissues in addition to tumor cells.

In our biodistribution studies in mice, there was low blood concentration and high tissue-to-blood contrast on three- and six-days post-injection. Notably, ^89^Zr-anti-CD44 showed remarkably high probe accumulation in the spleen. ^89^Zr-based PET imaging of CD44 was first explored in a study that used an anti-human Ab called RG7356, and demonstrated high uptake in human breast cancer xenografts of mice^7^. However, because RG7356 does not cross-react with murine CD44, it does not provide information regarding CD44 expression in physiologically normal organs of the host. ^89^Zr-labeled RG7356 was therefore further injected into monkeys as a cross-reactive species and revealed remarkably high uptake in the spleen^7^. A first-in-human trial of ^89^Zr-labeled RG7356 in patients with solid malignancies also demonstrated high splenic uptake that required co-injection of excess cold Ab to achieve tumor targeting^8,9^. These previous studies indicate that radiolabeled Abs raised against the constant domain of CD44 protein of the host species accumulate to high levels in the spleen. This includes antibodies against murine CD44 in mice and antibodies against human CD44 in monkey models or human subjects.

Because IM7 reacts with both human and mouse CD44, we expected ^89^Zr-anti-CD44 to target both human CD44-expressing tumors and host splenic tissue. However, even though SNU-C5 human colon cancer cells expressed CD44 protein, ^89^Zr-anti-CD44 uptake in SNU-C5 tumors was disappointingly low. The remarkably high uptake in the spleen indicates that the spleen acted as a sink organ, which took up the bulk of the administered ^89^Zr-anti-CD44 before it had a chance to accumulate in the tumor tissue. CD44 is expressed on leukocytes abundant in the spleen. Among splenic leukocytes, certain lymphocytes express CD44, and studies have shown that T lymphocyte effector function is activated by CD44^26,27^. The spleen also contains a large number of monocytes/macrophages and lower numbers of neutrophils that express high levels of CD44.

Given the high level of ^89^Zr-anti-CD44 uptake in the spleen, we dissected which types of cells were chiefly responsible for this finding. Leukocyte size and granule content can be assessed by forward and side scatter measurements on flow cytometry, respectively. Hence, lymphocytes are identified as small cells containing small granules, while monocyte/macrophages are identified as larger cells with granules^28–31^. On flow cytometry, splenic lymphocytes were separated into region R1 and splenic myeloid cells including monocyte/macrophages and neutrophils into region R2. That region R2 contained myeloid cells was confirmed by positive CD11b expression, an integrin receptor present on monocytes, macrophages, and neutrophils^32^. Functionally, CD11b regulates adhesion and migration of these cells^32–34^. Myeloid cells of region R2 also revealed high CD44 expression that was significantly greater than that exhibited by lymphocytes of region R1. Together, these finding indicate that myeloid cells are responsible for the high ^89^Zr-anti-CD44 accumulation by the spleen. In the murine spleen, there are up to fivefold greater numbers of monocyte/macrophages than neutrophils^35,36^. Therefore, monocyte/macrophages likely contributed to splenic ^89^Zr-anti-CD44 uptake to a significantly greater extent than that of neutrophils.

We next tested how CD44 expression and ^89^Zr-anti-CD44 uptake in splenic leukocytes were influenced by immune activation. Regulation of CD44 expression plays a critical role in modulating phagocyte adhesion, migration, and inflammation. LPS is a bacterial cell wall component that modulates inflammatory responses. In phagocytic cells, LPS has been shown to increase CD44 expression and activate downstream inflammatory responses^37^. In our study, when mice were treated with LPS, R2 splenic myeloid cells showed a substantial increase of CD44 expression, whereas R1 lymphocytes did not. We further tested the effects of LPS stimulation on cultured monocytic THP-1 and RAW264.7 cells and confirmed significant increase of CD44 expression. This is consistent with a previous study that observed LPS to increase CD44 expression in normal monocytes and THP-1 cells^38^. In our study, the increase of CD44 expression was accompanied by significant increase of ^89^Zr-anti-CD44 uptake. Furthermore, mice treated with LPS displayed significantly increased ^89^Zr-anti-CD44 uptake on PET images and biodistribution studies. Moreover, LPS treatment significantly increased ^89^Zr-anti-CD44 uptake in splenic myeloid cells of R2 but not splenic lymphocytes of R1. Taken together, our findings indicate that increased spleen uptake of ^89^Zr-anti-CD44 following LPS treatment represents upregulated CD44 expression on activated myeloid cells and largely on activated monocyte/macrophages.

Our results of splenic cell CD44 imaging has several clinical implications for immuno-PET using Abs raised against the constant domain of CD44 protein. For example, a better understanding of factors that influence anti-CD44 accumulation in non-tumor organs could help develop ways to increase CD44-targeted delivery to tumors^2–4^. In addition, the ability to image myeloid cell CD44 expression might assist in developing ways to intercept the splenic reservoir before their deployment to other sites^18^. Furthermore, imaging of myeloid cells may facilitate newer strategies to target the spleen for manipulating tolerance induction, tumor-induced myelopoiesis, and immune suppression therapies.

Finally, we attempted to improve tumor imaging with ^89^Zr-anti-CD44 by increasing the total dose of Ab injected. The reasoning behind this was that Ab dose can strongly affect biodistribution. Hence, elevated amounts of circulating Ab could partly saturate binding sites on splenic myeloid cells, thereby allow more ^89^Zr-anti-CD44 to reach the tumor for accumulation. The results revealed that administering ^89^Zr-anti-CD44 with a total Ab dose of 300 μg indeed reduced the level of splenic uptake and improved tumor uptake compared to when 100 μg was administered. Administering ^89^Zr-anti-CD44 with a total Ab dose of 700 μg further reduced splenic uptake half-fold and augmented tumor uptake two-fold. These results provide a potential method to improve tumor imaging with ^89^Zr-anti-CD44 PET.

In conclusion, using an ^89^Zr labeled Ab that cross-reacts with both human and mouse CD44, we show that CD44 immuno-PET has the capacity to monitor CD44 regulation on splenic myeloid cells and may also be useful for noninvasively imaging of tumors in mice by increasing Ab dose.

## Materials and methods

### Cell culture and reagents

THP-1 human monocytic cells and RAW264.7 murine macrophage cells were from the American Type Cell Culture. HT29 and SNU-C5 human colon cancer cells were from the Korea Cell Line Bank. Cells were maintained in 5% CO_2_ at 37 °C in RPMI-1640 media (Lonza, Basel, Swiss) supplemented with 10% fetal bovine serum (FBS; Serena, Germany), 2 mM L-glutamine, and 100 U/mL penicillin–streptomycin (Lonza, Basel, Swiss). Lipopolysaccharide (LPS) from Sigma Chemicals (St. Louis, MO) was dissolved in dimethyl sulfoxide (DMSO) and applied to cells by addition to culture medium.

IM7, a rat monoclonal Ab against human and mouse CD44 that reacts with all CD44 isoforms, was from Bio-X Cell (Lebanon, NH). Rabbit antibodies against CD44 were from Abcam (UK, Cambridge), and horseradish peroxidase-conjugated secondary anti-rabbit antibodies were from Cell Signaling Technology (Danvers, MA).

### Deferoxamine conjugation and site-specific ^89^Zr labeling of anti-CD44 Ab

IM7 was site-specifically conjugated with DFO-maleimide on sulfohydryl residues as previously described^23,24^. Briefly, 2 mg of anti-CD44 in a volume of 200 μl (10 mg/ml) was mixed with 100 mM TCEP (Sigma Chemicals) at a molar ratio of 1:100 and incubated for 20 min at room temperature. Reduced anti-CD44 was diluted in 0.1 M sodium phosphate containing 150 mM NaCl and 1 mM ethylene diamine tetraacetic acid (EDTA). The Ab was then conjugated on the sulfohydryl residues with 56.4 µL of 2 mM (0.44 μM) N-(3,11,14,22,25,33-hexaoxo-4,10,15,21,26,32-hexaaza-10,21,32-trihydroxytetratriacontane) maleimide (DFO-maleimide; Macrocyclics, TX) for 60 min at room temperature (RT). Ab-conjugated DFO was purified from unreacted DFO-maleimide by PD-10 column elution. ^89^Zr-oxalate (Korea Atomic Energy Research Institute) was neutralized with 2 M Na_2_CO_3_ and 74 MBq of ^89^Zr in 200 μl was mixed with DFO-conjugated anti-CD44 in 0.5 M HEPES buffer (pH 7.0). After 60 min of incubation with tapping every 15 min, the reaction mixture was eluted through a PD-10 column with 0.25 M sodium acetate containing 0.5% gentisic acid to purify ^89^Zr-anti-CD44 from free ^89^Zr and unconjugated DFO-maleimide. Collected fractions of 0.5 mL were counted on a high-energy γ-counter, and the peak activity fraction was used.

### SDS–polyacrylamide gel electrophoresis (PAGE) and autoradiography

For non-reducing SDS-PAGE, 2 µg of intact, TCEP-reduced, and DFO-conjugated anti-CD44 was diluted with water and 5 × non-reducing sample buffer without dithiothreitol (DTT). Samples were boiled at 95 °C for 10 min and then separated on an 8% sodium dodecyl sulfate (SDS) polyacrylamide gel by electrophoresis. The gel was subsequently stained with 0.5% Coomassie blue.

Autoradiography was also performed for ^89^Zr-anti-CD44 and was separated by 8% native PAGE with sample buffer free of SDS or DTT.

### Radiochemical stability assessment

^89^Zr-anti-CD44 was tested for radiochemical purity and stability by radio-iTLC as previously described^23,24^. ^89^Zr-anti-CD44 (15 μl of 1 mg/ml; 555 kBq) was incubated with the same volume of FBS or PBS at 37 °C. At 0, 1, or 4 days, 6 μl of the samples were placed on an iTLC-SG glass microfiber chromatography paper impregnated with silica gel (Agilent Technologies, CA). Radio-TLC was performed using an eluent of 50 mM EDTA (pH 5.5). In this condition, ^89^Zr-anti-CD44 remains at baseline, while free ^89^Zr^4+^ ions and [^89^Zr]-EDTA migrate at the solvent front. We did not investigate migration of ^89^Zr-DFO in this study.

### ^89^Zr-CD44 cell binding assays

Cultured SNU-C5, HT29, RAW264.7, and THP-1 cells or cells obtained from mouse spleen were counted and 0.25 × 10^6^ cells in 500 μl of culture media were placed into 1.5 mL tubes. Cells were incubated for 1 h with 185 kBq (0.36 μg) of ^89^Zr-anti-CD44 added to the media in 5% CO_2_ at 37 °C. After rapid washing twice with 500 μl of cold phosphate buffered saline (PBS), cells were measured for bound radioactivity on a high energy γ-counter (Perkin-Elmer). Excess cold Ab (500 nM) was used for blocking experiments. Uptake level of each sample was normalized for protein content.

### Western blotting for CD44 protein

Western blots were performed as previously described^24^. Briefly, cells were washed with PBS and solubilized in PRO-PREP protein extraction solution (iNtRON Biotechnology, Korea) for 15 min at 4 °C. Cell debris was eliminated by centrifugation at 14,000 rpm for 10 min at 4 °C. The supernatant was analyzed with Bradford protein assays, and 40 μg of protein was separated on a 10% polyacrylamide gel. The protein bands were transferred to a PVDF membrane (Amersham Biosciences; Piscataway, NJ) and incubated overnight at 4 °C with polyclonal antibodies against CD44 (1:1000) in Tris-buffered saline (50 mM Tris, pH 7.5, 150 mM NaCl) containing 0.05% Tween-20 and 5% skim milk. After washing 3 times for 10 min each with tris-buffered saline with Tween-20, the membrane was incubated with secondary antibodies at RT for 1 h. Immune reactive protein was finally detected with an enhanced chemiluminescence kit (Thermo Fisher Scientific, MA).

### Preparation of single-cell suspensions from mouse spleen

All animal experiments were performed in accordance with the National Institutes of Health Guide for Care and Use of Laboratory Animals and approved by the Institutional Animal Care and Use Committee of Samsung Medical Center. This study was carried out in compliance with the ARRIVE guidelines (http://www.nc3rs.org.uk/page.asp?id=1357). Single-cell suspensions of mouse spleen were prepared following a Stem Cell Technologies Protocol (https://www.stemcell.com/how-to-prepare-a-single-cell-suspension-from-mouse-spleen.html). Briefly, spleens extracted from normal 6-week-old male Balb/C mice sacrificed by cervical dislocation were minced in PBS by pressing with a syringe bar. The minced tissue solution was passed through a 100 µm mesh strainer (Corning, NY) using 2 × volume of PBS containing 2% FBS. Cell debris was removed by 3 min centrifugation at 1200 rpm. Red blood cells in the pellet were removed by 5 min treatment with RBC lysis buffer (10 mM Tris–HCl [pH 7.3] containing 140 mM NH_4_Cl and 1 mM EDTA), followed by rapid neutralization with PBS containing 2% FBS. The resultant single-cell suspension was finally washed twice with PBS containing 2% FBS and used for flow cytometry.

### Flow cytometry for CD11b and CD44 expression

RAW264.7 cells, THP-1 cells, or murine spleen cells (1 × 10^6^ cells) were incubated for 30 min at 37 °C with FITC-tagged anti-CD44 (BioLegend, CA, 1:200; 2.5 μg/ml) or APC-tagged anti-CD44 (BD Biosciences; 1:200; 2.5 μg/ml). The cells underwent FACS caliber flow cytometry analysis or FACS aria cell sorting (BD Biosciences). FITC fluorescence was detected with a 488 nm laser as excitation channel and 530 nm wavelength fluorescence as emission detector channel. APC fluorescence was detected by a combination of green 532-nm laser as excitation and 585/42-nm as the wavelength detector channel.

### ^89^Zr-anti-CD44 PET imaging and biodistribution

Pharmacokinetic studies, and LPS stimulation experiments with PET/CT imaging and biodistribution were performed in six-week-old male wild type Balb/C mice. Tumor-bearing models were prepared in six-week-old male Balb/C nude mice by subcutaneous injection of 5 × 10^6^ HT29 cancer cells or SNU-C5 into the right flank. Biodistribution and PET/CT imaging studies were performed in the mice when tumor diameter reached approximately 1.0 cm. All animals were injected into the tail vein with 3.7 MBq of ^89^Zr-anti-CD44 that contained an Ab dose of 100 μg, unless additional cold anti-CD44 was added to increase total Ab dose. Imaging was performed under isoflurane anesthesia using a small-animal PET/CT scanner (Inveon; Siemens) without respiratory gating. Acquisition of non-enhanced CT images was followed by emission PET imaging. Animals were sacrificed immediately after imaging, and tissues were weighed and measured for radioactivity on a high-energy γ-counter (Parkin Elmer). Uptake levels were expressed as percentage of injected dose per gram of tissue (%ID/g).

LPS stimulation was performed by daily intra-peritoneal injection of 50 μg LPS in saline for 3 days (saline was injected for controls). ^89^Zr-anti-CD44 was injected on the second day of LPS treatment, and PET imaging was performed 4 days later.

### Statistical analysis

Data are mean ± SD unless otherwise specified. Significant differences between two groups were analyzed by two-tailed unpaired Student’s t-test. For three or more groups, ANOVA with Tukey’s post-hoc test was used for comparison. *P* values < 0.05 were considered statistically significant.

## Supplementary information

Supplementary information 1.

Supplementary information 2.
