# Supplementary material for: 89Zr anti-CD44 immuno-PET monitors CD44 expression on splenic myeloid cells and HT29 colon cancer cells
Source: Sci Rep. 2021 Feb 16;11:3876. doi: 10.1038/s41598-021-83496-3 (PMC7887231; doi:10.1038/s41598-021-83496-3)
Supplement: Supplementary file 1 — Supplementary information 1. [file 41598_2021_83496_MOESM1_ESM.docx]

**Supplementary information**

**^89^Zr Anti-CD44 Immuno-PET Monitors CD44 Expression**

**on Splenic Myeloid Cells and HT29 Colon Cancer Cells**

**(Short title: CD44 Immuno-PET of Splenic Cells and Cancer Cells)**

Jin Won Park, PhD^1^¶; Kyung-Ho Jung, PhD^2,3^¶; Jin Hee Lee, BA^2,3^;

Seung Hwan Moon, MD^3^; Young Seok Cho, MD^3^; Kyung-Han Lee, MD^2,3^

^1^Scripps Korea Antibody Institute, Chuncheon-si, Gangwon-do, Korea, ^2^Department of Nuclear Medicine, Samsung Medical Center, Seoul, Korea, ^3^Department of Health Sciences and Technology, SAIHST, Sungkyunkwan University School of Medicine, Seoul, Korea

¶ These two authors contributed equally to this work

First Author-1: Jin Won Park, PhD.

Scripps Korea Antibody Institute, 1, Kangwondeahak-gil, Chuncheon-si, Gangwon-do, Korea

Tel: 82-33-250-8098; [zzic2y@naver.com](mailto:zzic2y@naver.com)

First Author-2: Kyung-Ho Jung, PhD.

Department of Nuclear Medicine, Samsung Medical Center, 50 Ilwon-dong, Gangnam-gu, Seoul, Korea

Tel: 82-2-3410-2649; Fax : 82-2-3410-2639; [jkhope0834@gmail.com](mailto:jkhope0834@gmail.com)

Corresponding Author (For reprints): Kyung-Han Lee, MD, PhD.

Department of Nuclear Medicine, Samsung Medical Center, 50 Ilwon-dong, Gangnam-gu, Seoul, Korea

Tel : 82-2-3410-2630; Fax : 82-2-3410-2639; [khleenm@naver.co](mailto:khleenm@naver.co)m

Keywords: CD44, PET, Spleen, Myeloid cells Macrophages, Lipopolysaccharide


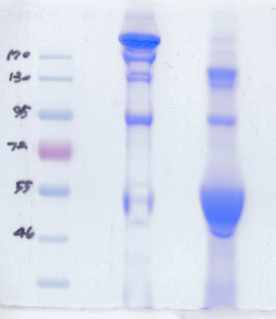

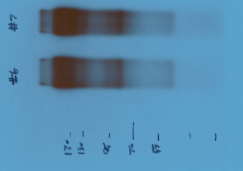


**Figure S1**. **Full length coomassie-stained gel and autoradiograph from figure 1A.** Commassie blue staining of samples loaded on non-reducing PAGE (left) and autoradiograph of ^89^Zr-CD44 Ab on native PAGE (right). Molecular weights are in kDa.


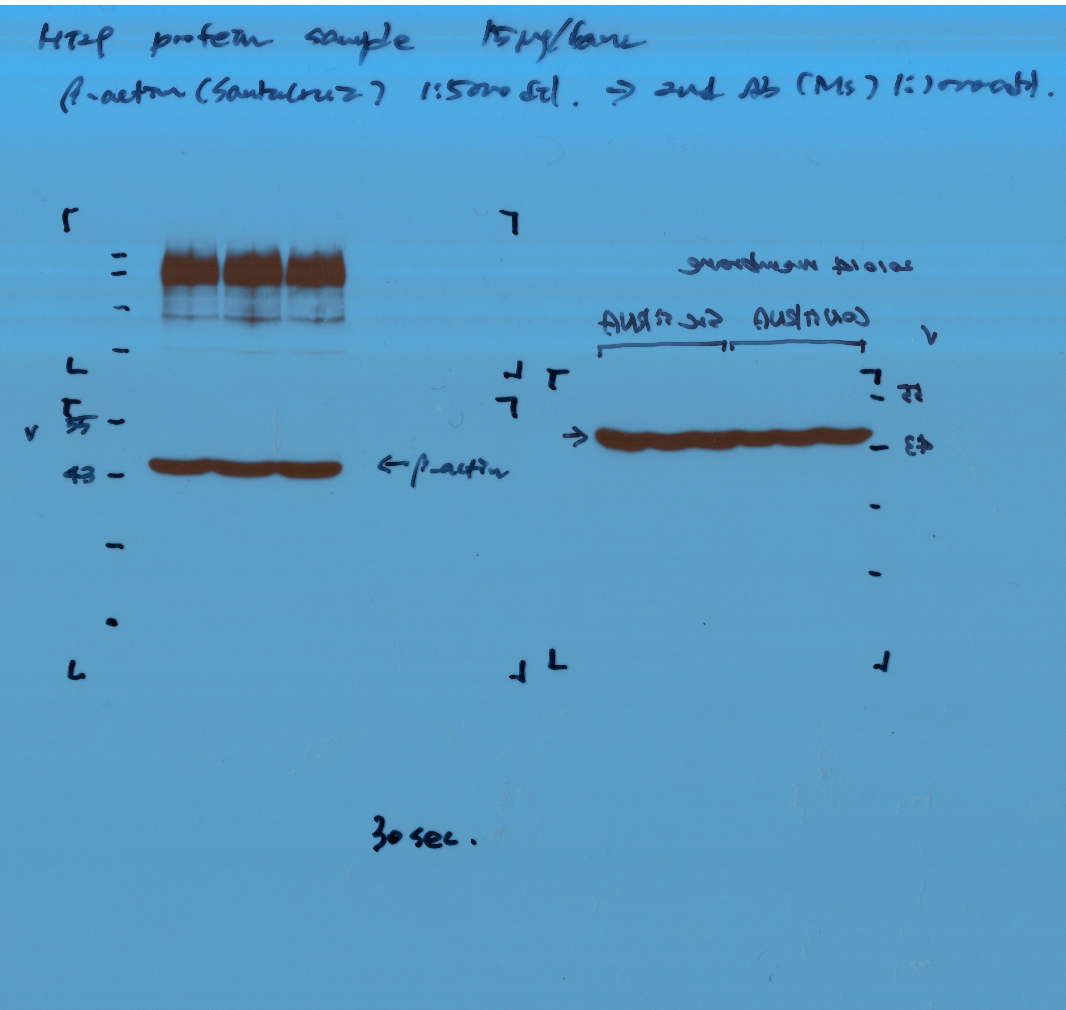

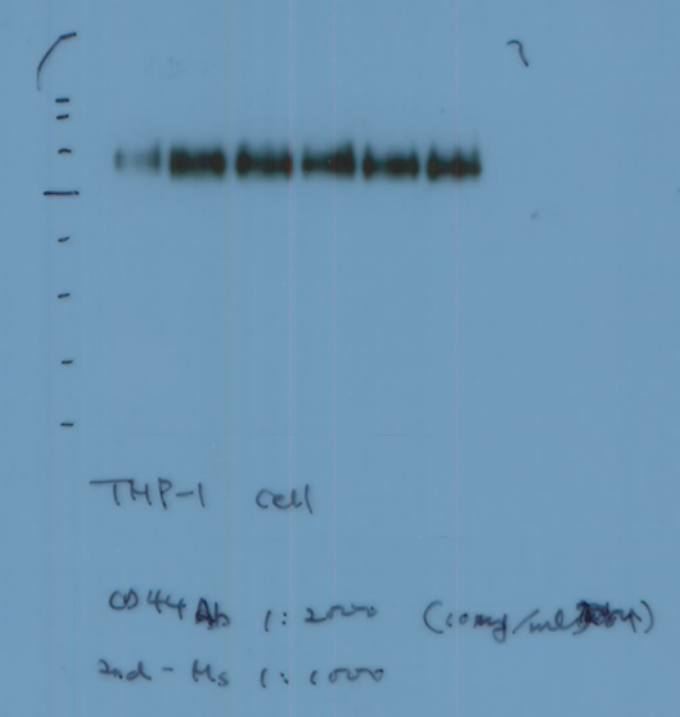


**Figure S2**. **Full length blots of cropped blots in figure 1B.** Western blots of protein from cancer cell lysates for detection of CD44 in HT29 (top), SNU-C5 (middle) and THP-1 cells (bottom). Molecular weights are in kDa.
