# Supplementary material for: 89Zr anti-CD44 immuno-PET monitors CD44 expression on splenic myeloid cells and HT29 colon cancer cells
Source: Sci Rep. 2021 Feb 16;11:3876. doi: 10.1038/s41598-021-83496-3 (PMC7887231; doi:10.1038/s41598-021-83496-3)
Supplement: Supplementary file 2 — Supplementary information 2. [file 41598_2021_83496_MOESM2_ESM.docx]

**SupplementARY TABLE 1**

^89^Zr-anti-CD44 biodistribution in tumor-bearing Balb/C nude mice

| **Organ or tissue** | 100 μg Ab dose  (n = 4) | 300 μg Ab dose  (n = 4) | 700 μg Ab dose  (n = 4) | *P* |
| --- | --- | --- | --- | --- |
| Blood | 0.12 ± 0.03 | 0.23 ± 0.03 | 2.29 ± 0.05 | *0.076* |
| Myocardium | 0.61 ± 0.18 | 0.40 ± 0.07 | 0.49 ± 0.02 | *0.061* |
| Lung | 0.86 ± 0.41 | 1.38 ± 0.52 | 1.75 ± 0.17 | *0.227* |
| Liver | 6.43 ± 0.99 | 5.69 ± 1.83 | 6.12 ± 1.06 | *0.700* |
| Spleen | 64.8 ± 8.02 | 25.15 ± 3.37 | 13.84 ± 1.44 | *0.000* |
| Stomach | 0.95 ± 0.29 | 1.11 ± 0.39 | 1.43 ± 0.15 | *0.174* |
| Kidney | 2.52 ± 0.53 | 2.68 ± 0.88 | 3.17 ± 0.44 | *0.353* |
| Muscle | 0.18 ± 0.08 | 0.14 ± 0.11 | 0.21 ± 0.03 | *0.249* |
| Tumor | 1.17 ± 0.68* | 2.88 ± 1.25** | 5.70 ± 0.70** | *0.007* |

*, SNU-C5 human colon cancer tumor in figure 1A; **, HT29 human colon cancer tumor in figure 6; Ab, antibody; *P* values are between 300 μg and 700 μg Ab dose groups
